# A novel mutation in the C-propeptide of *COL2A1* causes atypical spondyloepiphyseal dysplasia congenita

**DOI:** 10.1038/hgv.2017.3

**Published:** 2017-03-02

**Authors:** Chieko Kusano, Masaki Takagi, Naoaki Hori, Jun Murotsuki, Gen Nishimura, Tomonobu Hasegawa

**Affiliations:** 1 Department of Pediatrics, Ota Memorial Hospital, Gunma, Japan; 2 Division of Endocrinology and Metabolism, Tokyo Metropolitan Children’s Medical Center, Tokyo, Japan; 3 Division of Maternal and Fetal Medicine, Miyagi Children’s Hospital, Miyagi, Japan; 4 Department of Radiology, Tokyo Metropolitan Children’s Medical Center, Tokyo, Japan; 5 Department of Pediatrics, Keio University School of Medicine, Tokyo, Japan

## Abstract

Spondyloepiphyseal dysplasia congenita (SEDC, OMIM #183900) is one of the type II collagenopathies caused by a heterozygous mutation in the *COL2A1* gene. Although typical SEDC shows delay of pubic bone ossification on radiographs, atypical SEDC exists without this finding. We identified an atypical SEDC patient with a novel missense mutation in the C-propeptide region of *COL2A1*. This case suggests that a *COL2A1* C-propeptide mutation can cause atypical SEDC.

Spondyloepiphyseal dysplasia congenita (SEDC, OMIM #183900) is a bone dysplasia with autosomal dominant inheritance caused by a heterozygous mutation in the *COL2A1* gene (type II collagenopathy). Major clinical findings include disproportionately short stature with a short spine, short neck, barrel-shaped chest, flat face, myopia, cleft palate and hearing disorders. Respiratory distress at birth may occur because of a small thorax. Radiographs show platyspondyly with anterior wedging (pear-shaped), delayed ossification of the pubic bone and knee epiphyses, mild hypoplasia of iliac wings in infancy, hypoplasia of the odontoid process of C-2 and severe childhood presentations of coxa vara. These skeletal findings become milder with age. According to BIOBASE (http://www.biobase-international.com), over 100 *COL2A1* mutations have been reported to be associated with the SEDC phenotype. Barat-Houari *et al.*^1^ have recently reported that among SEDC patients, the most mutations occurs in the triple helix (74% glycine substitutions and 10% Arg-to-Cys changes), whereas a few other cases have been found to involve the C-propeptide region.

The *COL2A1* gene encodes the α1 chain of procollagen type II, which is the major fibrillar protein of hyaline cartilage, the vitreous humor of the eye and the disk of the inner ear. Three α1 chains fold together in a triple-helical configuration, thereby forming the procollagen homotrimer. The triple-helical region of the α1 chain is characterized by repeating Gly-X-Y triplets. Outside this triple helical region are the N- and C-propeptide regions. These regions are cleaved when during their exit from the endoplasmic reticulum. The C-propeptide region allows the initiation of the triple helical configuration. Thus, mutations in the *COL2A1* C-propeptide region can produce changes in chondrocyte morphology, collagen type II fibril structure and cartilage matrix composition^2^. Nishimura *et al.*^3^ have reported that the phenotypes of C-propeptide mutation in their series were atypical and included an absence of delayed pubic ossification. Here, we describe a female infant with atypical SEDC with a novel missense mutation in the C-propeptide region of the *COL2A1* gene.

A pregnant Japanese woman was referred to our hospital because of fetal ultrasound findings of short limbs at 28 weeks of gestation. She and her husband were healthy. Ultrasound revealed a short femur (−3.1 SD), and fetal CT scan showed ovoid vertebral bodies and mild iliac hypoplasia. A pubic bone ossification delay was not observed. The female baby was born at 36 weeks gestation through elective caesarean section. Her birth length was 40.6 cm (−3.7 SD), she weighed 2,360 g (−1.6 SD), and she had a head circumference of 34.0 cm (+1.0 SD). Her neonatal course was uneventful. Physical examination showed a short neck and short limbs. A bone radiograph at birth showed significant platyspondyly, shortening of long bones with ragged metaphyses, mild iliac hypoplasia and delayed ossification of femur heads. Because pubic bone ossification delay was not observed, we diagnosed this patient as having atypical SEDC (Figure 1). Ophthalmological examination did not reveal any abnormalities. The auditory brain stem response was unremarkable. At 2 years of age, her height was 69.0 cm (−5.8 SD) with a weight of 9.3 kg (−1.9 SD). She had a flat face and barrel-shaped chest. Bone radiography showed findings consistent with SEDC, such as pear-shaped vertebral bodies without pubic ossification delay. The pubic bone was normally ossified for her age. She did not develop myopia or hearing impairment.

We obtained written informed consent from her parents to perform molecular studies, which were approved by the Institutional Review Board of Keio University School of Medicine. Genomic DNA was extracted from the peripheral blood of the patient and her parents through standard techniques. We analyzed all coding exons and flanking introns of *COL2A1* by using PCR and direct sequencing. A cytosine to guanine substitution at position 3969 (c.3969c>g), resulting in an amino acid change of cysteine to tryptophan at position 1323 (p.C1323W) in exon 52 (Figure 2a), was identified in the patient, but not the parents. This variation has not been described in any other database, including dbSNP, the 1,000 Genomes Project, the Exome Variant Server, the NHLBI Exome Sequencing Project and the Human Genetic Variation Database in Japan. p.C1323 is conserved across various species (Figure 2b). *In silico* analysis showed that p.C1323W was predicted to be ‘probably damaging,’ with a score of 1.000, by PolyPhen-2 and ‘deleterious,’ with a score of 0.00, by SIFT.

p.C1323 is located in the C-propeptide region of the *COL2A1* gene. As Zabel *et al.*,^2^, Unger *et al.*^4^ and Richards *et al.*^5^ have reported, cysteines are critical residues within the C-propeptide region. They build intra- and intermolecular disulfide bonds, thereby mediating protein folding, trimer formation, chain registration and the initiation of helix formation. Furthermore, Bae *et al.*^6^ have reported a type II collagenopathy with p.C1323Y, thus indicating an important role of p.C1323 within the C-propeptide region. Altogether, we inferred that p.C1323W was a pathogenic mutation.

Nishimura *et al.*^3^ provided the first report that 2 atypical SEDC families with *COL2A1* C-propeptide mutations have characteristic findings in X-rays, such as an absence of pubic bone ossification delay. Nishimura et al. have stated that Unger *et al.*^4^ have previously reported an SEDC family with a *COL2A1* C-propeptide mutation. X-ray findings showed that the patients presented with an absence of pubic bone ossification delay in infancy. Similarly, our patient did not show pubic bone ossification delay. This is the 4th reported case of atypical SEDC with a *COL2A1* C-propeptide mutation presenting without pubic bone ossification in infancy. However, definitive correlations between genotype and phenotype in the *COL2A1* C-propeptide mutation and atypical SEDC currently remain unclear, and it is inconclusive whether 2 cases reported by Tarhal *et al.*^7^ with *COL2A1* C-propeptide mutations had delayed pubic bone ossification.

The frequency of hearing impairment as a complication of *COL2A1* C-propeptide mutations should be further investigated. Two out of 6 cases reported by Tahal *et al*. had complaints of hearing loss, whereas Nishimura *et al.*^3^ have reported that hearing impairment is infrequently observed in patients with mutations in this region. Our patient did not experience hearing impairment until 2 years of age. In conclusion, our case suggests that a *COL2A1* C-propeptide mutation can cause atypical SEDC.

## Figures and Tables

**Figure 1:**
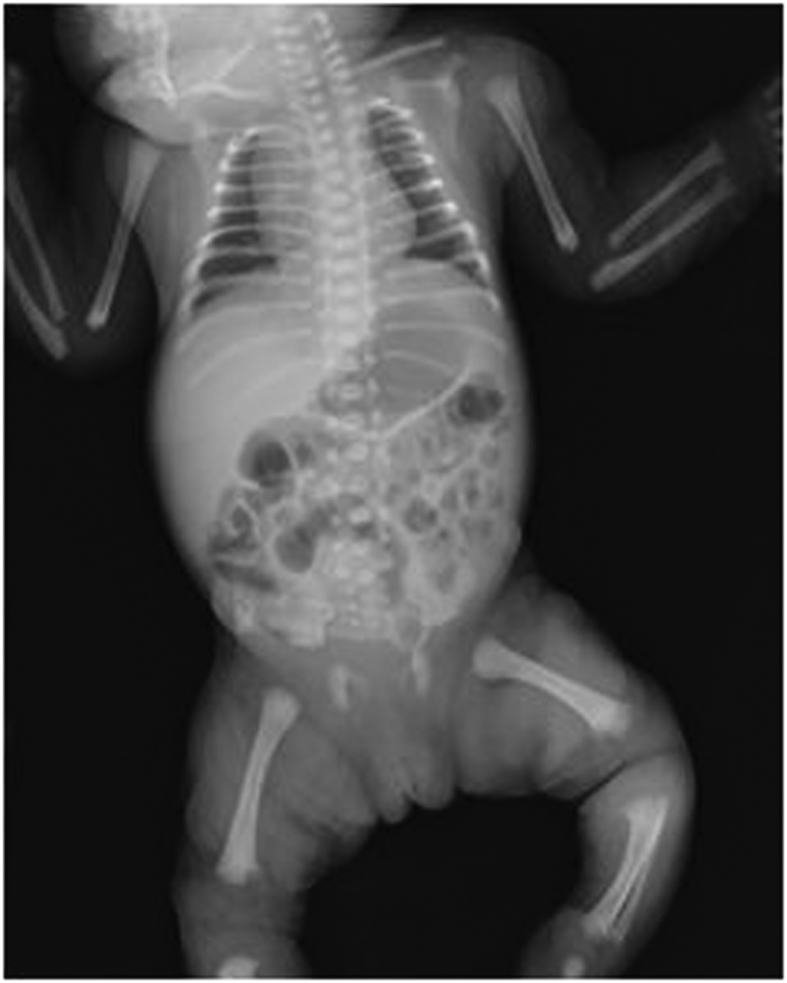
Radiograph of patient at birth. It showed typical findings such as significant platyspondyly, shortening of long bones with ragged metaphyses, mild iliac hypoplasia, and delayed ossification of femur head with absence of pubic ossification delay.

**Figure 2:**
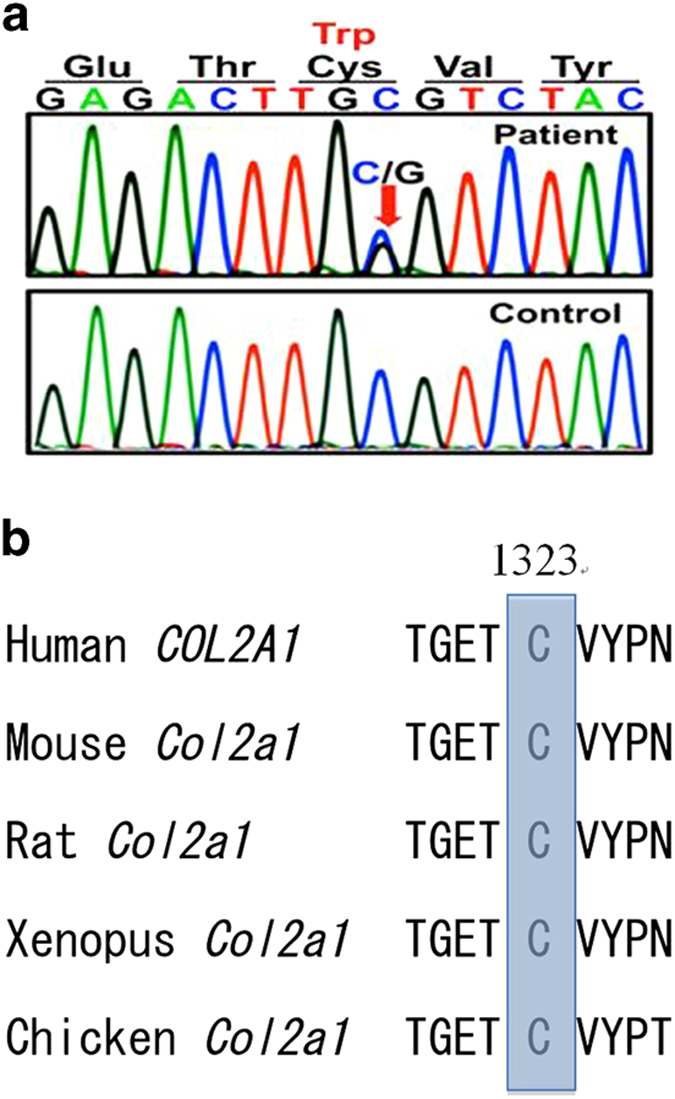
Results of the *COL2A1* gene analysis. (**a**) Partial chromatograms of *COL2A1* A substitution from cytosine at 3969 in the *COL2A1* cDNA to guanine (c.3969 c>g) that results in an amino acid change from cysteine at 1323 in the type II collagen α1-chain to tryptophan (p.C1323W) was identified in this patient. (**b**) Alignment of partial sequence of human *COL2A1* protein with four protein sequences. Cysteine at position 1323 in *Homo sapiens* is conserved in various species.
